# Suspension physics govern the multiscale dynamics of blood flow in sickle cell disease

**DOI:** 10.1126/sciadv.adx3842

**Published:** 2026-01-01

**Authors:** Hannah M. Szafraniec, Freya Bull, John M. Higgins, Howard A. Stone, Timm Krüger, Philip Pearce, David K. Wood

**Affiliations:** ^1^ Department of Biomedical Engineering, University of Minnesota, Minneapolis, MN, USA.; ^2^ Department of Mathematics, University College London, London, UK.; ^3^ Center for Systems Biology and Department of Pathology, Massachusetts General Hospital, Boston, MA, USA.; ^4^ Department of Systems Biology, Harvard Medical School, Boston, MA, USA.; ^5^ Department of Mechanical and Aerospace Engineering, Princeton University, Princeton, NJ, USA.; ^6^ School of Engineering, Institute for Multiscale Thermofluids, University of Edinburgh, Edinburgh, UK.; ^7^ Institute for the Physics of Living Systems, University College London, London, UK.

## Abstract

From diabetes to malaria, altered blood flow contributes to poor clinical outcomes. Heterogeneity in red blood cell (RBC) properties within and across individuals has hindered our ability to establish the multiscale mechanisms driving pathological flow dynamics in such diseases. To address this, we develop microfluidic platforms to measure RBC properties and flow dynamics in the same blood samples from patients with sickle cell disease (SCD). We find that effective blood viscosity across individuals is explained by the proportion of stiff RBCs, exhibiting qualitative similarities to rigid-particle suspensions, despite considerable mechanical heterogeneity. By combining simulations with spatially resolved measurements of cell dynamics, we show how features of emergent rheology are governed by spatiotemporal cell organization, via margination at intermediate oxygen tensions, and localized jamming caused by spatial hematocrit variations under hypoxia. Our work defines the suspension physics underlying pathological blood flow in SCD and, more broadly, emergent rheology in heterogeneous particle suspensions.

## Teaser

Cell mechanical heterogeneity is established as a main driver of aberrant blood flow across diverse patient profiles.

## INTRODUCTION

A common feature of cancer (*1*), metabolic diseases such as diabetes (*2*), and infectious diseases such as malaria (*3*) are pathological changes to the properties of blood, which can affect microvascular flow and disrupt nutrient and oxygen transport throughout the body. Sickle cell disease (SCD), which affects millions of people worldwide and 300,000 infants born each year, is a prevalent example of a genetic blood disorder (*4*). The pathology of SCD derives from a mutation in the oxygen-carrying hemoglobin molecule (*5*), causing it to aggregate into a polymer under deoxygenation, so that the red blood cells (RBCs) become significantly less deformable (*6*–*10*). The result is that patients with SCD exhibit pathological and variable blood flow, with disease progression and severity correlated with rheological and hemodynamic biomarkers, such as elevated viscosity and the increased likelihood of vaso-occlusive crises (*11*–*17*). Despite the prevalence of such blood disorders, we still lack a mechanistic understanding of how disease-driven alterations in the properties or distribution of blood cells alter hemodynamics and contribute to disease-associated vascular complications. For example, it remains unclear why individuals with SCD exhibit highly variable blood rheology, which is linked to certain clinical complications (*18*, *19*). We do not understand how the presence of stiff RBCs affects local blood flow in the microvasculature, where most of the hemodynamically driven disease processes, such as vaso-occlusive crisis, are thought to occur (*20*). Obtaining these insights has been difficult, and progress is limited by the lack of a unified single cell– and whole blood–level experimental platform capable of single-cell analysis, precise oxygen control, and spatially resolved flow measurements. By contrast, extensive work understanding healthy blood flow has aided progress in drug delivery, medical device designs, and clinical risk assessment in diseased vascular structures, such as aneurysms (*21*–*23*). Similar progress in blood disorders has been hampered by a lack of experimental measurements that directly connect disease-driven heterogeneity in RBC properties to emergent blood flow dynamics and effective rheology under physiological conditions.

Despite intensive research on the rheology of blood and of particle suspensions in general, established physical models are not able to connect cell and whole-blood properties in diseases such as SCD, in which blood contains subpopulations of highly stiff and highly deformable RBCs (*10*, *24*). Multiple studies using macroscale measurements with rheometers or simulations have shown that increasing the volume fraction of stiffened RBCs increases effective blood viscosity (*25*–*27*). Similarly, it is well established that increases in cell stiffness at a fixed volume fraction raise viscosity (*28*–*30*). These measurements provide a clear link between cell stiffening and increased blood viscosity, but they are difficult to interpret mechanistically and extrapolate to physiological hemodynamics. Specifically, measurements using rheometers are not spatially resolved, minimizing boundary effects and preventing them from capturing the microscopic dynamics of the cells, such as the well-known Fahraeus-Lindqvist and Fahraeus effects, which arise from interactions between cells and confining walls (*31*, *32*). How such phenomena are affected by heterogeneous RBC properties is not captured by macroscale measurements of effective viscosity. Although models have been developed that aim to accurately predict blood flow dynamics in confinement, they typically do so with the assumption that all cells are homogeneous and highly deformable (*33*–*37*). More generally, suspensions of homogeneous rigid particles or cells have been well characterized experimentally, theoretically, and computationally (*38*–*42*). These studies revealed that effects arising from the suspended particles themselves are considerable and, for example, lead to radial variations in particle concentration that cause apparent non-Newtonian rheological behavior, such as slip and shear thinning, in confined flow (*43*, *44*). Thus, both experiments and modeling in homogeneous suspensions, such as suspensions of RBCs or rigid particles, have revealed that microscopic effects, owing to the cells or particles, contribute to suspension rheology and flow dynamics. However, established relationships that link these bulk properties to particle or cell volume fractions do not account for the mechanical heterogeneity present under conditions like SCD. We expect heterogeneous mixtures of RBCs to have unique microscopic dynamics and macroscopic properties, owing to the stiff and deformable cells, but it remains unclear how these phenomena affect effective rheology and local flow dynamics (*45*–*47*). As a result, we do not understand how sickle blood flows under physiologically relevant conditions, including the microvasculature, where complications are likely to occur.

In this study, we advance our understanding of SCD hemodynamics under physiological conditions by mechanistically linking the distribution of RBC mechanical properties with spatially resolved blood flow, both transverse and along the flow directions, in confined geometries. We use a high-throughput imaging method to measure distinct subpopulations of RBCs within individual patient blood samples (*24*, *48*). In parallel, we measure the effective rheology of the blood derived from spatially resolved flow fields (*49*). To explore mechanisms leading to the emergent rheology, we develop a computational model to simulate the confined flow of heterogeneous mixtures of cells. This allows us to probe the dynamics of the cells and measure the impact on rheology, which we confirm experimentally. We are therefore able to directly link single-cell properties to blood dynamics in microchannels across individual donors and to establish mechanisms for the apparent rheology of heterogeneous mixtures. Our findings reveal how suspension physics govern the flow properties of sickle blood and explain differences between patients. Despite the presence of both stiffened and deformable cells in the blood, we find that our measurements are broadly consistent with previous empirical models for the rheology of rigid-particle suspensions across a range of particle volume fractions. In addition, our imaging and computational modeling reveal how overall dynamics are driven by complex spatiotemporal processes in mixtures of stiff and deformable cells. Therefore, our work elucidates the dominant physical parameters and mechanisms contributing to the pathological rheology for patients with SCD. More broadly, our findings advance the understanding of the flow of particle suspensions by revealing how rheological properties emerge from heterogeneity in particle properties.

## RESULTS

### Stiff RBCs drive altered blood rheology across oxygen tensions and among patients

**Fig. 1.**
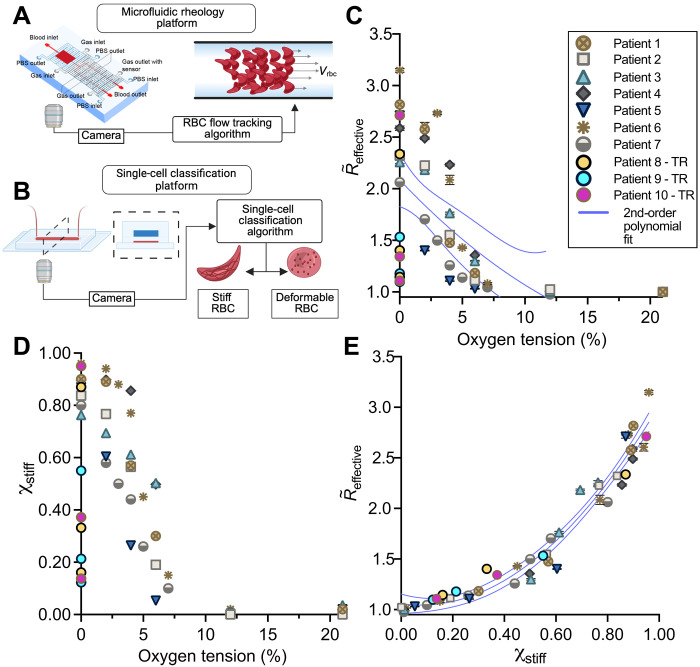
Stiff cells drive the increase in effective resistance for sickle blood under hypoxia and reduce interpatient variability. (**A**) Microfluidic rheology platform used to measure spatially resolved blood flow dynamics. Blood was perfused in a 20- by 20-μm cross-sectional channel, and the effective flow resistance was measured from flow data. (**B**) Microfluidic single-cell classification platform used to measure the RBC population distribution of stiff (cells with polymer) and deformable (cells without polymer) cells at various oxygen tensions. (**C**) Effective resistances were normalized by the 21% oxygen value for each patient and plotted against oxygen tension as ${\tilde{R}}_{\text{effective}}$. Results show unique patient response curves at oxygen tensions less than 12%. Patients 8, 9, and 10 were transfused twice, and measurements were performed at 0% oxygen, including the native sample. The variance measured by the SSE of a second-order polynomial curve fit was 0.28. (**D**) Stiff cell fractions plotted for each patient across a range of oxygen tensions. (**E**) ${\tilde{R}}_{\text{effective}}$ plotted against the stiff cell fraction decreased interpatient rheological variability by a factor of 17 with a variance of 0.020. Results shown for *n* = 10 sickle blood samples. Error bars represent ±SEM for resistance data gathered for *n* = 11 independent sampling time points during flow data acquisition. Error bars smaller than the data symbol are not shown. Statistical analysis performed in Prism version 9.5.0 using nonlinear least-squares regression of a second-order polynomial. Second-order polynomial fits (blue line) are plotted with 95% confidence bands (dashed lines). Illustrations created in BioRender. Szafraniec, H. (2025) https://biorender.com/cg83txe.

Using single-cell measurements in parallel with a microfluidic rheological platform, we combined measurements of cell properties—the fraction of RBCs that are stiff because of hemoglobin S polymerization—with whole-blood dynamics—cell tracks, effective flow fields, and effective flow resistance (${\tilde{R}}_{\text{effective}}$, i.e., the ratio of pressure drop to flow rate normalized to the 21% oxygen value). We performed experiments over a range of imposed oxygen tensions within the same samples from multiple patients (Fig. 1, A and B, and “Blood sample collection and preparation,” “Blood preparation,” “Microfluidic device manufacturing,” “Microfluidic rheology platform, measurement system, and flow rate validation,” and “Single-cell microfluidic platform” sections). We hypothesized that the proportion of stiff RBCs drives the rheological response to hypoxia. Given that our blood flow experiments have a fixed volume fraction of RBCs (Φ *=* 25%), our results are scaled to the fraction of RBCs that are stiff ($\chi_{\text{stiff}}$), where $\chi_{\text{stiff}}=\Phi_{\text{stiff}}/\Phi$. While both ${\tilde{R}}_{\text{effective}}$ and $\chi_{\text{stiff}}$ increased with decreasing oxygen, each patient exhibited a unique response curve (Fig. 1, C and D). By plotting ${\tilde{R}}_{\text{effective}}$ against $\chi_{\text{stiff}}$, we found that all samples collapsed onto a single curve, including patient rheology from exchange transfusion treatment (Fig. 1E and “Blood preparation” section). Therefore, for a fixed volume fraction of RBCs in suspension, these results indicate that the fraction of stiff RBCs is the key microscopic driver of whole-blood rheology across patients with SCD and over the full physiological range of oxygen tensions.

### Whole blood from patients with SCD behaves macroscopically as a suspension of rigid particles with effective properties

**Fig. 2.**
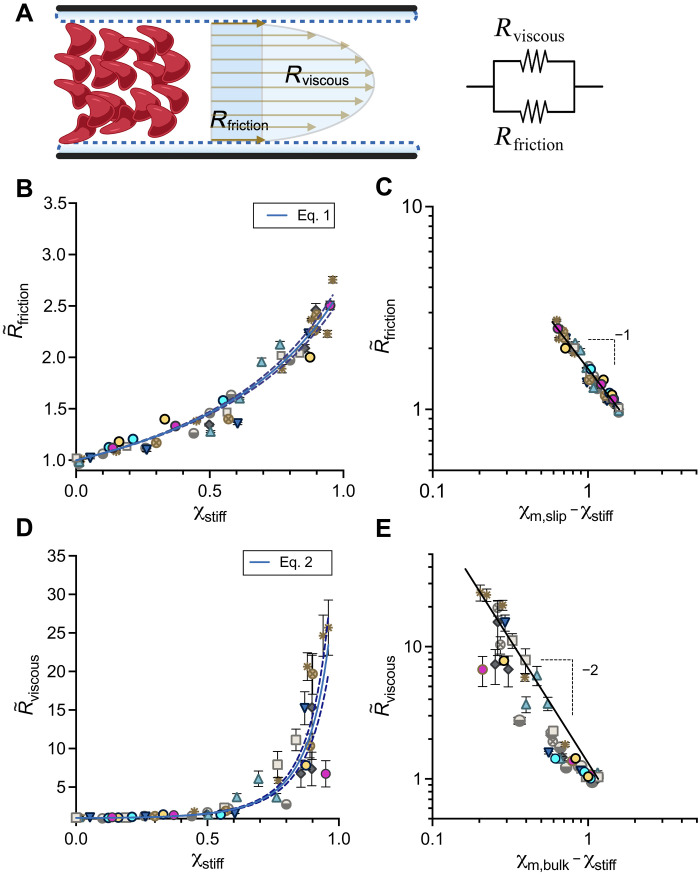
Critical microvascular rheology of SCD blood compared to theory in rigid-particle suspensions. (**A**) Local velocity fields in the microfluidic channel are obtained using high-speed imaging and feature tracking methods to generate flow profiles. Flow profiles approximate the height-averaged flow, which is used to measure the effective rheology. With a fixed pressure drop, the frictional resistance, measured from the slip flow rate, and viscous resistance, measured from the bulk flow rate, combine in parallel to form the effective resistance. (**B**) ${\tilde{R}}_{\text{friction}}$ plotted against $\chi_{\text{stiff}}$ and fit with Eq. 1. (**C**) The logarithmic plot of ${\tilde{R}}_{\text{friction}}$ versus ($\chi_{m,\text{slip}}-\chi_{\text{stiff}}$) compared to Eq. 1 (black line) shows good qualitative agreement between data and model for a $\chi_{m,\text{slip}}$ of 1.59 (95% confidence interval: 1.56, 1.62). (**D**) ${\tilde{R}}_{\text{viscous}}$ versus $\chi_{\text{stiff}}$. Data fit with Eq. 2. (**E**) The logarithmic plot of ${\tilde{R}}_{\text{viscous}}$ versus ($\chi_{m,\text{bulk}}-\chi_{\text{stiff}}$) compared to Eq. 2 (black line) shows good qualitative agreement between data and model for a $\chi_{m,\text{bulk}}$ of 1.16 (95% confidence interval: 1.15, 1.18). Results shown for *n* = 10 sickle blood samples. Error bars represent ±SEM for resistance data gathered for *n* = 11 independent sampling time points during flow data acquisition. Error bars smaller than the data symbol are not shown. Equations 1 and 2 (blue lines) are fit to the data in (B) and (D), respectively, using a nonlinear least-squares regression performed in Prism version 9.5.0. and plotted with 95% confidence bands (dashed line).

Next, we explored the physical basis for the relationship between stiff cell fraction and effective flow resistance. Using the motion of the cells, we constructed flow fields and resistances using the material model of an effective fluid with an apparent slip velocity and effective bulk viscosity (*50*), where the total flow resistance of the blood is due to the resistance to slip or effective wall friction (*R*_*friction*_), associated with the velocity of the cells near the channel walls, and the bulk viscosity (*R*_*viscous*_), which describes the remaining resistance to flow owing to the effective bulk properties of the blood (Fig. 2A and “Microfluidic rheology platform, measurement system, and flow rate validation” section). Briefly, *R*_*friction*_ *=* ∆*P*/*Q*_slip_ and *R*_*viscous*_ *= ∆P/Q*_bulk_, where *∆P* is the pressure drop across the experimental channel, *Q*_*slip*_ is the slip flow rate, and *Q*_*bulk*_ is the bulk flow rate. The values of *R*_*friction*_ [0.0091 ± 0.0013(SD) Pa·s μm^−3^] and *R*_*viscous*_ [0.037 ± 0.011(SD) Pa·s μm^−3^] at 21% oxygen demonstrate the contribution of slip and bulk viscosity on the overall resistance in the absence of stiffened cells (fig. S1). We then normalized each resistance, *R*_*friction*_(O_2_) and *R*_*viscous*_(O_2_), to the corresponding resistance measured at 21% oxygen, *R*_*friction*_ (21%) and *R*_*viscous*_ (21%), respectively. We used these normalized resistances, defined as ${\tilde{R}}_{\text{friction}}$ and ${\tilde{\text{R}}}_{viscous}$, to represent macroscopic measurements of the overall oxygen-dependent effective rheology of the blood and to evaluate the effects resulting from stiffened cells (Fig. 2, B to E).

In suspensions of homogeneous rigid particles, a range of experimental measurements has demonstrated that slip and bulk resistances follow established empirical relationships on the basis of the volume fraction of the particles. For suspensions of low-aspect-ratio particles, the slip length has been measured as a function of particle diameter, particle volume fraction, and critical particle volume fraction (*39*). Here, we apply this relationship in Eq. 1 considering ${\tilde{R}}_{\text{friction}}$ as an equivalent measurement of the inverse slip length and substitute the volume fraction of rigid particles for the directly measured quantity**,** $\chi_{\text{stiff}}$. We also assume the cell diameter to be fixed. ${\tilde{R}}_{\text{friction}}$ is thus fit with Eq. 1 using the single parameter $\chi_{m,\text{slip}}$

$${\tilde{R}}_{\text{friction}}\left(\chi_{\text{stiff}}\right)=1+\frac{\chi_{\text{stiff}}}{\chi_{m,\text{slip}}-\chi_{\text{stiff}}}$$ (1)

The second feature, viscous resistance, measures the bulk viscosity of the suspension. The bulk viscosity is defined by the volume fraction of particles; however, the relationship is highly dependent on the volume fraction regime. In the dilute limit, the bulk viscosity is linear in the volume fraction (*51*, *52*). As volume fractions increase, the relationship becomes nonlinear and a divergence in the viscosity occurs as the suspension approaches a jamming transition (*40*). Here, we apply this relationship in Eq. 2 considering ${\tilde{R}}_{\text{viscous}}$ as an equivalent measurement of bulk viscosity, with $\chi_{\text{stiff}}$ substituted for volume fraction, using a single parameter, $\chi_{m,\text{bulk}}$

$${\tilde{R}}_{\text{viscous}}\left(\chi_{\text{stiff}}\right)=1+{\left(\frac{\chi_{\text{stiff}}}{\chi_{m,\text{bulk}}-\chi_{\text{stiff}}}\right)}^{2}$$ (2)

We find that the frictional resistance and viscous resistance scale with the fraction of stiff cells in a similar manner to homogeneous suspensions with increasing volume fraction of rigid particles despite the overall inflow volume fraction of RBCs being constant in all our experiments. For the frictional resistance, we find the entirety of the data range to be well described by Eq. 1, and we note that increases in ${\tilde{R}}_{\text{friction}}$ occur for even small amounts of stiff cells (Fig. 2, C and D). We also find ${\tilde{R}}_{\text{viscous}}$ to be well described by Eq. 2 (Fig. 2, E and F). However, given our estimated inflow blood volume fractions of 25%, the divergence in ${\tilde{R}}_{\text{viscous}}$ seems to occur at a lower volume fraction than predicted from rigid-particle suspension theory (fig. S2). Overall, our results suggest that blood containing stiff RBCs obeys rigid-particle suspension mechanics. However, it is not yet clear what determines the effective parameters describing the critical volume fractions in the well-known empirical relationships.

### In simulations, margination of stiff RBCs increases frictional resistance by increasing the local volume fraction of stiff cells near channel walls

**Fig. 3.**
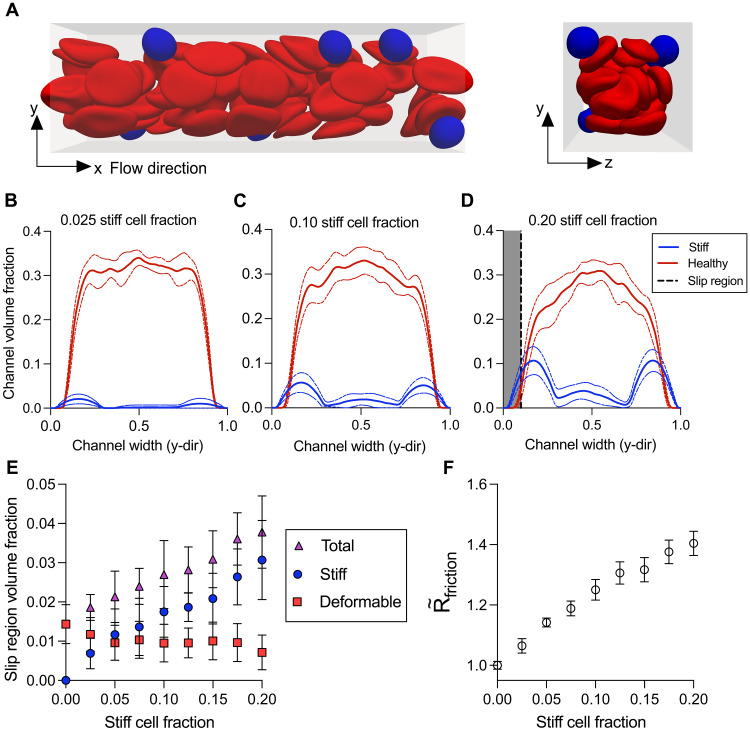
Simulations of heterogeneous blood suspensions in confined flow. (**A**) Simulation data show spatial localization of the stiff cells (blue) near the channel boundaries compared to the deformable (red) cells. Local channel volume fractions of the stiff and deformable cells across the channel width for stiff cell fractions of (**B**) 0.025, (**C**) 0.10, and (**D**) 0.20. Mean (solid line) and 95% confidence interval (dashed line) plotted for local channel volume fractions of stiff (blue) and deformable (red) cells for *n* = 10 simulations. Given the finite resolution of the simulations, a slip region (gray), rather than slip length, was defined as the region within 10% of the channel width to the wall and held constant. (**E**) The mean volume fraction of stiff (blue circle), deformable (red square), and total (purple triangle) cells within the slip region for each stiff cell fraction is plotted. Error bars representing ±SEM for *n* = 10 simulations. (**F**) Normalized mean frictional resistance (open circle) plotted against stiff cell fraction. The frictional resistance was calculated using the pressure drop and the slip flow rate, quantified by the average velocity in the slip region. Error bars representing ±SEM for *n* = 10 simulations.

The increases in friction occurring at relatively low stiff RBC fractions suggest extensive interactions between the stiff cells and the channel wall. We hypothesized that complex interactions between deformable and stiff cells could account for this (*53*). Therefore, to explore mechanisms for the increases in frictional resistance, we simulated the flow of RBC suspensions composed of mixtures of deformable and stiff cells using a computational model. In our simulations, a lattice-Boltzmann method was used for fluid flow, a finite-element method for cell dynamics, and an immersed boundary method for fluid-structure interactions (*36*, *54*, *55*). Cells were immersed in a Newtonian fluid and confined to a rectangular channel with flow driven by a constant body force, mimicking a fixed pressure gradient. We performed simulations parameterized from experimental conditions corresponding to a 25% volume fraction of total cells, a 20- by 20-μm cross-sectional area, and the appropriate pressure drop per unit length (“RBC simulation methods” section). The simulated stiff cell fraction was modulated by increasing the number of stiff cells while decreasing the number of deformable cells. Qualitatively, the simulated suspension demonstrates margination of the stiffer cells toward the channel boundaries (Fig. 3A). Analysis of the local volume fraction of stiff and deformable cells in the simulations shows an increase in volume fraction of stiff cells near the channel walls as the stiff cell fraction increases (Fig. 3, B to E). Furthermore, by performing resistance analyses on the simulations, we found that this effect drives an increase in ${\tilde{R}}_{\text{friction}}$ (Fig. 3F).

### In experiments, margination of stiffened RBCs results in rapid localization near channel walls

**Fig. 4.**
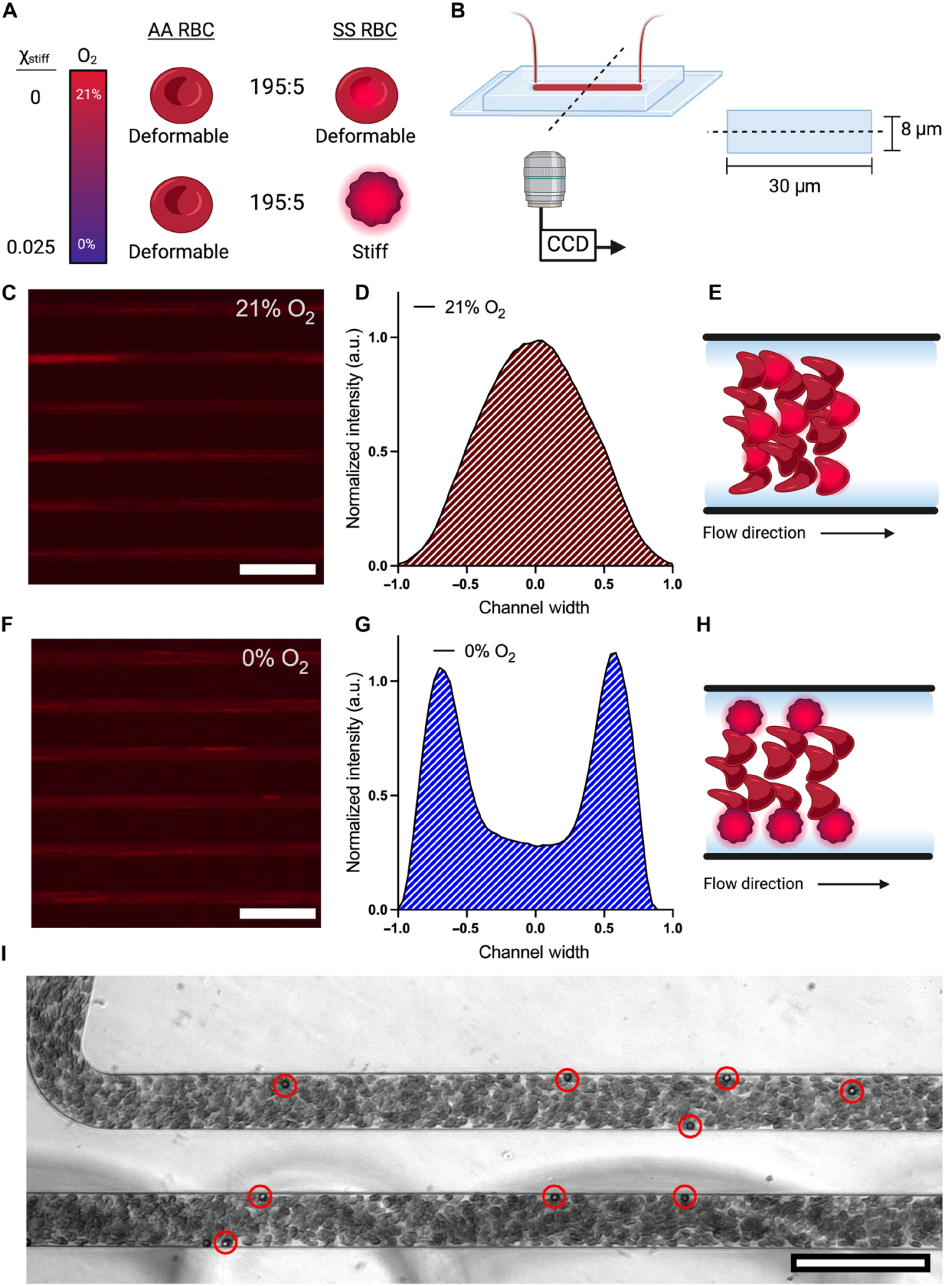
Sickle RBCs marginating in response to hypoxia when present in mixtures of healthy RBCs. (**A**) RBCs from a donor without SCD (AA RBCs) are mixed with stained sickle RBCs (SS RBCs) at volume ratios of 195:5. Under 21% oxygen, all cells are deformable. Therefore, the fluorescent signal represents deformable RBC flow. Under 0% oxygen, the sickle RBCs stiffen and provide a fluorescent signal for the spatial distribution of the cells. (**B**) Schematic of the microfluidic channel geometry for blood flow. Images were captured at a distance of 10 mm from the inlet of the device, approximately half the length of the channel used for the rheological studies. (**C**) Representative fluorescence images of cells flowing in the microfluidic device under 21% oxygen. Images taken at 5× magnification show 6 of the 21 channels in the device. (**D**) Spatial distribution of fluorescent signal under 21% oxygen for SS RBCs. a.u., arbitrary units. (**E**) Representative drawing of RBC flow arrangement in heterogeneous mixtures when all cells are deformable. (**F**) Representative fluorescence image of cells flowing in the microfluidic device under 0% oxygen. Images taken at 5× magnification show 6 of the 21 channels in the device. (**G**) Spatial distribution of fluorescent signal under 0% oxygen for SS RBCs. (**H**) Representative drawing of RBC flow arrangement when the SS RBCs are stiff because of hypoxia. (**I**) Experimental image of 5-μm-diameter polystyrene beads marginating in healthy blood. Red circles highlight beads for clarity. Scale bars represent 100 μm. Data shown for a single representative blood sample. Illustrations created in BioRender. Szafraniec, H. (2025) https://biorender.com/cg83txe.

To test the predictions from the simulations experimentally, we fluorescently stained RBCs from SCD donors and mixed the stained sickle RBCs with unstained RBCs from donors without SCD to a final volume fraction of 25% (Fig. 4A and “Fluorescent cell staining protocol and analysis” section). Upon deoxygenation, only the stained cells become stiff, and the fluorescent signal provides their spatial location. The cells were perfused through a microfluidic device composed of 21 channels, 30 μm wide and 8 μm tall, under a fixed pressure drop of 250 mbar (Fig. 4B). This design provided a quasi–two-dimensional configuration in which margination occurs in the imaging plane, and the fluorescent signal is enhanced by eliminating any out-of-focus effects. Representative images of the fluorescent signals from the stained cells under 21 and 0% oxygen are shown in Fig. 4 (C and F, respectively). The fluorescent streaks represent stained sickle RBCs (streaking is due to the long exposure times needed) (movies S1 and S2). The spatial distribution of the fluorescent signal under 21% oxygen shows a distribution centered around the middle of the channel, suggesting that the sickle RBCs concentrate in the middle of the channel when they are deformable (Fig. 4D). However, under deoxygenation, the sickle RBCs concentrate near the boundaries, indicating mechanically induced margination (Fig. 4G). Together, these experimental results confirm the predictions from our simulations that margination, as a result of heterogeneity in mechanical properties, changes the spatial organization of cells and drives the increase in frictional resistance at low stiff cell fractions. Furthermore, we sought to understand the kinetics of margination as it relates to timescales relevant to in vivo conditions. Therefore, we seeded 25% RBC volume fraction healthy blood with 5-μm-diameter bovine serum albumin (BSA)–coated polystyrene beads at a 0.1% volume fraction. We observed rapid margination occurring at the inlet of the microfluidic device. Most beads marginated in the first channel and appeared to concentrate near the channel corners (Fig. 4I and movie S3). Therefore, we expect margination to occur rapidly in microfluidic flows and contribute to the local rheology measured in sickle blood flow with low stiff cell fractions.

### Axial variations in local volume fraction drive extreme profile blunting at high stiff cell fractions

**Fig. 5.**
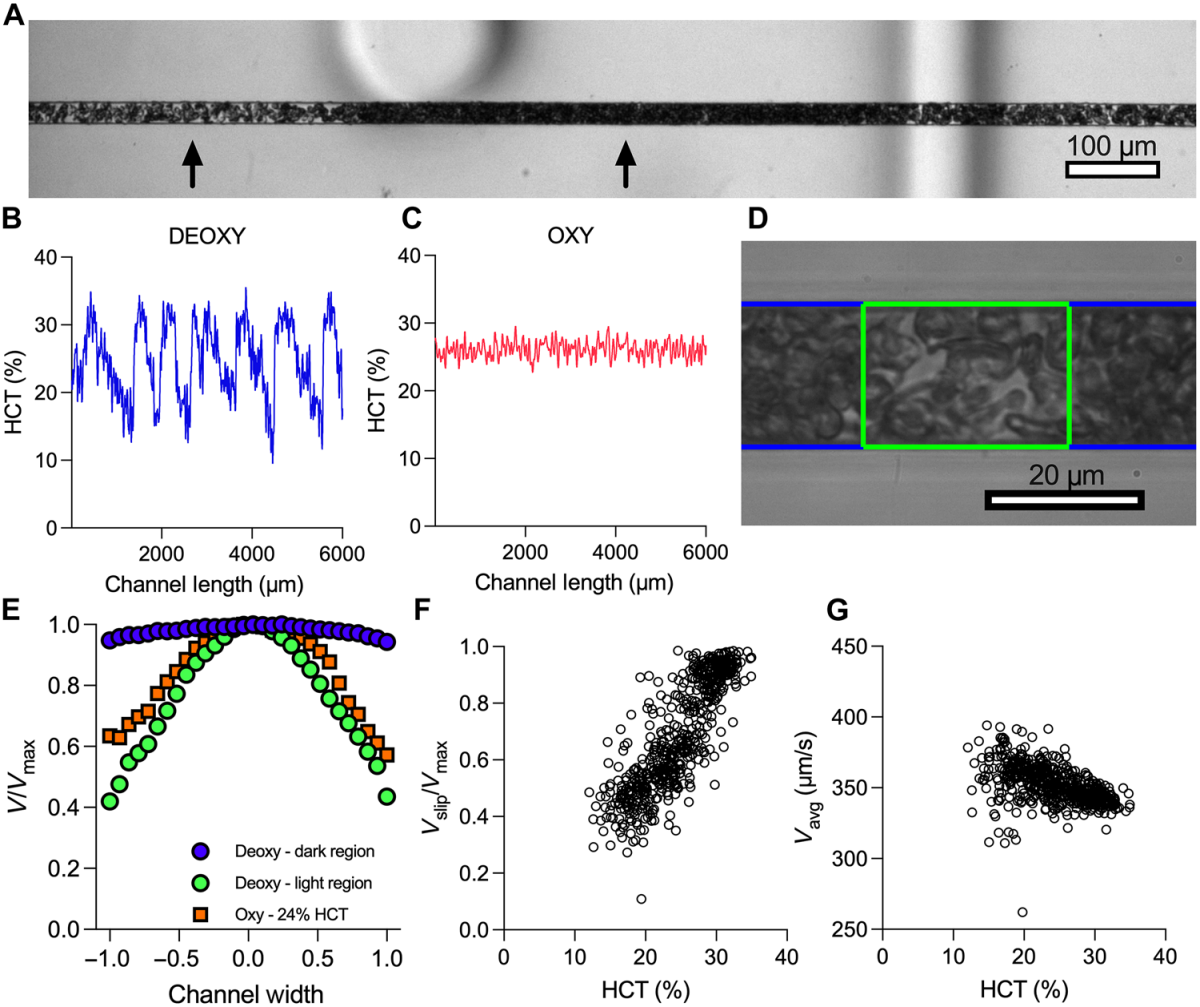
Axial variations in local RBC volume fractions and extreme profile blunting occur at high stiff cell fractions. (**A**) Axial variations in the local RBC volume fractions in the microfluidic channel for SCD blood under 0% oxygen. The bright region has a low RBC concentration, while the dark region has a higher RBC concentration. Regions are noted by arrows for clarity. (**B**) The local HCT was quantified over the length of the experimental channel. Oscillations in the local HCT fluctuated between 12 and 35%. (**C**) Local HCT across the length of the channel at 21% oxygen. (**D**) Local flow data are chunked into high-HCT (blue) and low-HCT (green) regions. Local flow profiles within each region are measured and compared to the corresponding HCT. (**E**) Normalized flow profiles from the high-HCT region (blue circle) and low-HCT region (green circle) are plotted and compared to 21% oxygen (orange square). The normalized flow profiles reveal that extreme blunting occurs in the higher-HCT region. (**F**) *V*_*slip*_*/*V**_*max*_ from chunked data is plotted against the corresponding HCT. (**G**) Average speeds from the chunked data plotted against corresponding HCT. Data in [(B), (C), and (E) to (G)] were obtained from a single representative SCD blood sample.

At high values of the stiff RBC fraction, we found that the viscous resistance diverges like that of a concentrated suspension approaching jamming (Fig. 2D). However, the inflow RBC volume fractions, or hematocrit (HCT), used in our experiments were well below the theoretical jamming volume fraction of 58 to 64% for rigid particles (*42*). To obtain a clearer understanding of the overall blood dynamics in this scenario, we obtained experimental videos of flowing blood over extended length and timescales (Fig. 5A and movie S4). At low oxygen tensions, variations in the local volume fractions of RBCs along the length of the channel developed, as seen by changes in local light intensity (Fig. 5A). Under fully deoxygenated conditions, we estimated the local HCT that corresponds to high- and low-intensity regions from video data (“HCT estimation protocol and validation” section). The results for blood from a representative donor demonstrate that the HCT is no longer uniform along the length of the channel, with values as high as 35% and as low as 12% and a mean HCT of 24% (Fig. 5B). This finding contrasts with the local HCT appearing relatively uniform at 21% oxygen (Fig. 5C). Using the local gradients in the axial HCT signal, we segmented the video data by HCT and measured the local flow features as a function of the local HCT (Fig. 5D). We found the normalized velocity profiles in the high-HCT regions to show extreme profile flattening. By contrast, the velocity profiles in the low-HCT regions were more parabolic (Fig. 5E). For a representative sample, we found that the profile bluntness, quantified by the slip velocity divided by the maximum velocity (*V*_*slip*_*/*V**_*max*_), clearly depends on the local HCT (Fig. 5F). Meanwhile, the average local velocity (*V*_*avg*_) varied to some extent with HCT; however, these findings were expected given the spatiotemporal heterogeneity in HCT as well as *V*_*avg*_ representing only the average speed of the solid phase (Fig. 5G). In summary, our findings reveal that the overall blood flow dynamics measured at high stiff cell fractions are a result of axial variations in the local volume fractions of cells, which can be observed as temporal fluctuations in volume fraction. Given the extreme blunting in the high-HCT regimes, these regions demonstrate an apparent jamming behavior, driving the increases in the macroscopic resistances like that of a concentrated suspension.

## DISCUSSION

Here, we developed a microfluidic platform along with computational modeling to measure and understand how heterogeneity in RBC properties drives blood rheology. Our results show that the extreme variability in blood rheology among individuals with SCD, which is known for heterogeneity in RBC properties, is almost entirely explained by accounting for the distribution of stiff cells in suspension at a given oxygen tension. We find that these mechanically heterogeneous mixtures obey rigid-particle suspension mechanics on a macroscopic level. However, the mechanisms driving the rheological behavior are highly dependent on the cross-sectional and axial spatial distribution of cells. Ultimately, these findings establish cell mechanical heterogeneity as the key biophysical mechanism that can explain pathological changes in blood rheology in SCD and capture patient-level differences. This crucial insight into how stiff RBCs disrupt blood flow in SCD may also inform our understanding of other hematologic disorders marked by altered or heterogeneous RBC properties. More broadly, we reveal the multiscale processes that determine emergent rheology in heterogeneous particle suspensions.

### Suspension rheology

Our findings have implications in suspension rheology—blood from patients with SCD is a heterogeneous suspension containing a mixture of stiff and deformable cells, with the relative number of each cell type determined by the oxygen tension in a patient-specific way. Our spatially resolved measurements allow us to extract the overall flow resistance of such suspensions in microchannels and to decompose the overall resistance into a frictional component, determined by wall slip, and a viscous component, determined by bulk viscosity. We find that all three of these resistances are largely determined by the fraction of stiff cells in the mixed suspension. However, there are important spatial complexities in suspension organization, which determine how the flow resistances depend on stiff cell fraction. For example, at low stiff cell fractions, we see a cross-sectional variance in cell organization—heterogeneity in cell mechanical properties drives margination and localization of stiff cells near channel walls. This leads to a reduction in the cell-free layer thickness, and therefore an increase in frictional resistance, even for very small numbers of stiff cells. This finding contrasts with suspensions of homogeneous rigid particles, where classic theories do not consider frictional effects at very low particle volume fractions in confinement (*51*, *52*). Furthermore, in more recent measurements of apparent slip in rigid particle suspensions (*56*, *57*), decreases in particle-free layer thicknesses are observed only above 20% volume fraction of particles (*39*, *58*). By contrast, our results show increases in frictional resistance at stiff cell fractions less than 0.20, corresponding to volume fractions less than 5.0%. These results demonstrate the combined importance of particle organization and geometric confinement in determining the flow behavior of heterogeneous particle suspensions.

Our measurements also have implications in homogeneous suspension rheology under confinement. At high stiff cell fractions, which occur under full deoxygenation, the suspension becomes mechanically homogeneous. In this regime, the increases in effective resistances are broadly consistent with prior studies in soft particle suspensions, which find that increasing cell stiffness shifts the jamming volume fraction closer to the hard-sphere case, thus raising the viscosity at a fixed volume fraction (*28*, *30*). However, in addition to this homogeneous contribution, we also find that the effective viscosities are further increased by axial heterogeneity in the local volume fraction of cells; in particular, regions of high and low volume fractions develop with distinct flow profiles. We observe the extreme bluntness of the flow profiles in the high-volume fraction regions, which qualitatively reflects an apparent jamming behavior in the bulk portion of the suspension (*59*). Despite the local packing, the volume fractions in these regions are substantially lower than classical measurements of the jamming fraction for suspensions of rigid particles (*40*, *42*). We suspect that additional effects such as cell shape, confinement, or cell roughness may be responsible. Suspensions of particles with nonspherical shapes with aspect ratios near unity can exhibit a decreased jamming fraction (*60*) but not enough to fully explain our findings [we did not observe high aspect ratios in our cells, a finding consistent with previous studies (*10*, *61*, *62*)]. Similarly, recent theory and simulations show that particle roughness can generate effective friction between cells, leading to an earlier divergence in viscosity at lower volume fractions (*63*–*65*). Effects due to confinement may also play a role: Cross-sectional variations in particle density can contribute to flow profile blunting as local concentrations approach the jamming fraction; detecting such effects typically requires advanced imaging techniques with an improved lateral resolution (*44*). Together, our findings suggest a complex interplay of all these effects—shape, roughness, and confinement—in determining flow resistance under fully deoxygenated conditions. Broadly, our findings highlight the importance of spatial particle organization in determining the rheology of suspensions in confined geometries, which provides a framework for incorporating predictions of jamming fractions made from bulk viscosity measurements to a confined, axially heterogeneous flow (*66*).

### Pathophysiology

Here, we demonstrate hypoxia-induced margination of unaltered RBCs from SCD donors. Given that we detect stiff cells at physiological oxygen tensions as high as 12% oxygen, our results suggest that small amounts of these stiff cells are likely present throughout the vasculature and that margination of these cells occurs even at low abundance. This corroborates the findings of previous work that stiffened RBC margination in SCD is an important biophysical contribution to systemic vascular inflammation and disease pathology (*47*). Building on this, our findings complement previous work regarding the effects of margination on endothelial inflammation and local shear stress fluctuations, as well as margination efficiency, by explaining how margination of stiffer RBCs affects flow profiles and frictional resistances in particular (*46*, *47*, *67*). We establish that the process of margination of stiff RBCs increases near-wall resistance, counteracting the Fahraeus effect and thereby increasing the overall effective resistance by increasing the concentration of stiffened cells near channel walls in the microvasculature. We postulate that small amounts of stiffened cells may have a pronounced effect on peripheral vascular resistance, which is reduced in healthy blood by wall effects, and their presence near blood vessel walls may alter mechanical homeostasis in the vasculature (*68*). Further work should be aimed at understanding the dynamics of these small populations of stiff cells in more complex vascular networks to provide insight into their impact on network-level flow dynamics, possibly indicating which vascular structures might be most susceptible to margination-induced inflammation, and to help guide more complex in vivo studies (*69*). To obtain direct quantitative agreement with experiments in such simulations will require careful calibration of cell material properties (*70*) on the basis of emerging experimental measurements (*71*). The physiological implications of our results demonstrating axial-temporal variation in HCT under extreme hypoxia are less clear. However, we note that such axial variation has been observed in blood flow imaging in the vasculature of transgenic sickle mouse models (*72*) and patients with SCD (*73*). Thus, we conclude that this is not an experimental artifact but a real phenomenon, and more work is warranted to understand the potential clinical impact.

### Clinical relevance and outlook

Overall, we show that the hypoxia-driven rheological responses across multiple blood donors appear remarkably similar when accounting for the distribution of cells with polymerized hemoglobin. In previous analyses of blood from patients with SCD, a clear physical understanding of how suspension physics govern blood rheology has remained elusive because of technical hurdles, as well as the complex physiochemical processes that govern cell properties. These processes, which include oxygen binding and molecular aggregation during deoxygenation (*6*), are influenced by patient-specific differences in cell size, hemoglobin content, and ongoing disease treatments (*7*, *48*); in turn, these cell-level differences lead to patient-specific whole-blood properties (*19*, *74*). Here, by integrating single-cell and whole-blood measurements, we establish quantitative links between cellular properties and blood rheology, accounting for patient-level differences and responses to medical interventions such as exchange transfusion therapy, which markedly alter the fraction of stiffened RBCs. In addition, therapies such as hydroxyurea or gene therapy, which increase fetal hemoglobin as a therapeutic strategy, aim to modulate patient-specific stiff RBC distributions, however with unpredictable and variable efficacy (*48*). Therefore, our findings establish a mechanistic link between therapeutic targets, the stiff cell fraction, and clinically, correlated measurable aspects of the patient-specific blood rheology. These findings further motivate the need for single RBC measurements for assessing and optimizing therapeutic strategies for individuals with SCD (*75*). Beyond SCD, diseases such as malaria and diabetes also compromise the mechanical deformability of the RBC, and the mechanisms driving increases in blood viscosity are attributed to increases in RBC stiffness (*76*, *77*). Evaluating pathological rheology as it relates to heterogeneity in the properties of RBCs is likely relevant for a range of diseases, and the results here provide a framework to understand how pathologies may evolve across those diseases.

## MATERIALS AND METHODS

### Blood sample collection and preparation

Blood samples from healthy donors and donors with SCD were collected at the Massachusetts General Hospital under Institutional Review Board (IRB)–approved protocols (2006P000066) and at the University of Minnesota and Children’s Hospital and Clinics of Minnesota under IRB-approved protocols (STUDY00003). All human individuals gave informed consent before participating in this study, or a waiver of consent was approved by the IRB after a determination of minimal risk. Complete blood counts and hemoglobin variant fractions HbA, HbS, HbA2, and HbF are included in table S1.

### Blood preparation

All blood samples used for experiments were collected in 0.109 M sodium citrate buffer (BD, cat. no. 363083) and stored up to 4 days at 4°C before testing. For rheological measurements, blood plasma was removed using a three-step wash and centrifugation procedure at 400*g*. Washed RBCs were resuspended in 2% bovine serum albumin (Sigma-Aldrich, cat. no. A7030-50G) in Dulbecco’s phosphate-buffered saline (PBS) (Corning, cat. no. 21-031-CV) to a target of 25% HCT or RBC volume fraction. The HCT target was chosen on the basis of the average HCT for patients with SCD. For single-cell measurements, 12 μl of packed, washed RBCs was added to 288 μl of 25% human serum albumin solution (Gemini Bio, cat. no. 800-120) and 12 μl of acid blue 9 (0.8 g/dl) in 1× PBS (AB9, TCI America, Portland, OR; cat. no. B0790). For exchange transfusions, washed healthy donor blood (HbA-only blood) was suspended to a target HCT of 25% in 2% BSA and mixed at different volume ratios with 25% HCT HbS blood. Samples chosen for transfusion were type matched and had similar mean corpuscular volume values. Packed RBC subsamples of these mixtures were used for single-cell measurements.

### Microfluidic device manufacturing

All experimental studies were conducted using polydimethylsiloxane (PDMS) devices. Briefly, the microfluidic rheology device consists of three layers bonded together. The first layer is composed of two gas chambers, 150 μm tall, allowing for independent control of oxygen to either side of the device. The second layer is a 100-μm-tall hydration layer. The third layer is composed of a 40- by 20-μm resistor that bifurcates into two 20- by 20-μm channels, 22 mm in length. The single-cell microfluidic device was manufactured using the exact design from Di Caprio *et al.* (*24*). The margination microfluidic device design was adapted from the design of Di Caprio *et al.* using a blood layer composed of 21 channels, each 8 μm tall, 30 μm wide, and 40 mm long. Silicon wafer molds were fabricated using negative resist photolithography for the indicated feature geometries. Each PDMS layer was fabricated by soft lithography from silicon wafer molds. The PDMS elastomer and curing agent were mixed using a 10:1 ratio by weight (Sylgard 184, Dow Corning, US). Microfluidic layers were cured at 75°C for 2 hours, bonded on the same day, and used within 24 hours of manufacturing. Each layer was plasma bonded at 10 cc/min air flow rate, 75% power, and 60-s exposure time settings (PE-50, PlasmaEtch) and cured at 120°C for 5 min. The merged layers were then plasma bonded to a clean glass microscope slide using the same plasma settings and cured at 120°C for 5 min.

### Microfluidic rheology platform, measurement system, and flow rate validation

Using a microfluidic platform previously described, flow measurements were made in a 20- by 20-μm channel under varying degrees of hypoxia (*18*, *49*).To characterize each blood sample, the microfluidic rheology device was visualized on a Zeiss Axio Vert microscope using a 40× objective [Zeiss 40×/0.6 numerical aperture (NA), air] encased in a 37°C environmental incubator chamber. Blood was perfused at a fixed pressure drop and initial average velocity of 900 μm/s. Images were acquired using a high-speed camera (Phantom Miro C-110) at 400 fps for a 512-by-1280 image at a region of interest near the end of the experimental channel. Oxygen (21% O_2_, balance N_2_) was cycled from 21% to the desired hypoxic condition by mixing with pure nitrogen using a solenoid valve gas mixer reported previously (*18*, *19*). Blood rheology was measured by directly visualizing the blood flow and calculating local velocity profiles using computer vision techniques. Briefly, the total RBC flow rate, *Q*_*total*_, in the experimental channel was calculated, assuming that the velocity profile corresponds to a height-averaged velocity profile across the device; therefore, *Q*_*total*_ = *V*_*avg*_ × width × height. During 21% oxygen conditions, the experimental and bypass channels are assumed to have the same flow rate. A flow meter (Flow Unit XS, Fluigent) was then incorporated into the microfluidic system to validate flow rates measured using computer vision techniques. We found no significant difference between the flow meter reading and computer vision estimates at different time points corresponding to 21% oxygen during our experiments when both channels have the same flow rate (fig. S3). Effective friction and viscous resistances were calculated from the flow rates using methods described previously (*49*). Briefly, *R*_friction_ = ∆*P*/*Q*_slip_ and *R*_*viscous*_ = *∆P*/*Q*_bulk_. *∆P* is the pressure drop across the experimental channel, *Q*_*slip*_ is the slip flow rate, and *Q*_*bulk*_ is the bulk flow rate. *Q*_*slip*_ is calculated from the slip velocity, and *V*_*slip*_ is multiplied by the channel cross-sectional area. To obtain *V*_*slip*_, we followed the previously reported methods (*49*). Briefly, we bin the spatial *x* velocities into 30 bins in the *y* direction. The velocities in the outermost bins, those closest to the wall, are averaged to obtain *V*_*slip*_. *Q*_*bulk*_ is simply the difference between *Q*_*total*_ and *Q*_*slip*_. Here, we normalized the resistance calculations to the corresponding 21% oxygen value immediately preceding the deoxygenation cycle step so that any effects due to temporal drift were minimized.

### Single-cell microfluidic platform

RBCs containing HbS polymer at various oxygen tensions were identified using a high-throughput single-cell microfluidic platform following the methods previously described (*24*). Experiments were run on the same day as the blood rheology experiments. Blood was prepared as described in the “Blood preparation” section. Prepared blood was visualized using a Zeiss Axio Vert microscope with a 40×/0.75-NA air objective encased in a 37°C environmental incubator chamber. Images of single RBCs at three different wavelengths are captured on a color camera (Flir Blackfly S). 610 nm was used to measure RBC volume by dye exclusion, and 405 and 430 nm were used to quantify the amount of oxy and deoxy hemoglobin on the basis of the relative absorbance at each wavelength. The RGB (red, green, and blue) images were then classified as either stiff (containing HbS polymer) or deformable (no polymer) using a Resnet50 algorithm previously validated and tested (*48*). Population distributions of at least 1000 cells were used to estimate stiff cell fractions ranging from 0 to 1 for each oxygen tension.

### Data analysis, statistics, and reproducibility

Data were fit to a *n*-order polynomial using the least-squares regression method. The order of polynomial was chosen as the lowest value in which the *r*-square value was minimal without overfitting the data and maintaining a monotonic form. The curve fit (solid lines) and 95% confidence intervals (dashed lines) were plotted. The sum of square errors (SSE) was then used to compute the variance, defined as SSE/(*n* − 1), and compared between plots. Statistical analysis was performed in Prism version 9.5.0. All experimental measurements used in this study were taken from different blood samples. For simulations, 10 simulations were run for each condition. Error bars represent plus or minus the standard error of the mean (±SEM) for *n* = 10 simulations. The mean (solid line) and 95% confidence interval (dashed line) are plotted for local channel volume fraction estimates. Flow data were analyzed in MATLAB 2022b. Single-cell data were analyzed in MATLAB 2021a and Python 3.10.11.

### RBC simulation methods

We performed simulations using a previously developed numerical model: a hybrid immersed-boundary lattice-Boltzmann finite-element model (*78*). The suspending fluid was solved by a lattice Boltzmann solver for a geometry of 64 μm by 20 μm by 20 μm (288-by-90-by-90 grid points), periodic in the *x* direction, with a viscosity of 0.001 Pa·s and an imposed pressure gradient of 1.3 × 10^5^ N/m^3^ (compare to an experimental pressure gradient of 1.44 × 10^5^ N/m^3^). Healthy RBCs were represented by a finite-element triangular mesh with 6480 facets, with the equilibrium shape as a biconcave disk with a radius of 18 grid points (4 μm). The membrane model of the RBC was as by Krüger *et al.* (*54*), parameterized by (in simulation units) (κ_*S*_, κ_*B*_, κ_α_, κ_*A*_, κ_*V*_) = (0.00132, 0.00107, 0.75, 1, 0.75), corresponding to healthy RBCs with a shear modulus of 5 × 10^−6^ N/m. Stiff RBCs were represented by a triangular mesh with 2880 facets, with the equilibrium shape as a sphere with a radius of 12 grid points (~2.67-μm; enclosed sphere volume is ~80% of healthy cell enclosed volume), parameterized by (κ_*S*_, κ_*B*_, κ_α_, κ_*A*_, κ_*V*_) = (0.0132, 0.00474, 0.75, 1, 0.75), corresponding to a shear modulus 10 times greater than the healthy RBC shear modulus (5 × 10^−5^ N/m). In our simulations, we fixed the cell volume fraction as 25%, resulting in simulations run with the number of stiff:healthy cells as (0:63, 2:61, 4:60, 6:58, 8:57, 10:55, 12:53, 14:52, 16:50). To improve numerical stability and avoid particle overlap, a short-range repulsive force between cells was used. This force was zero for distances larger than 2 grid points and behaved as 1/*r*^2^ for shorter distances. To improve statistics, all simulations were repeated 10 times, with random initial RBC configurations. Statistics were calculated after the simulation had been run for 1.0 × 10^6^ time steps (equivalent to 0.5 s) to ensure that a quasi-steady state had been attained (verified by confirming the convergence of calculated statistics). Cell volume distributions were calculated by finding the enclosed cell volumes bounded by parallel planes located at *y* = 0 and *y* = *Y*, for *Y* in [0,90], at a time step of 10^6^. The frictional resistance was defined as 1/*V*_*slip*_ normalized by the frictional resistance for the zero stiff cell case (100% deformable cells). *V*_*slip*_ is the slip velocity, defined as the mean *x* velocity of the nodes with *y* in grid point boundaries [0,9) or (81,90] (within 10% channel width of the wall) for 10^6^ < *t* < 1.6 × 10^6^.

### Fluorescent cell staining protocol and analysis

RBCs from an untransfused SCD blood sample were fluorescently stained by pipetting 5 μl of whole blood from sodium citrate tubes into 995 μl of a 1/1000 dilution of CellMask DeepRed (C10046) in 1× PBS. The blood stain mixture was incubated for 5 min and spun at 400*g* for 3 min, and the supernatant was removed. Stained RBCs were resuspended in 1 ml of PBS and then spun at 400*g* for 3 min. The supernatant was removed, and the remaining pellet was resuspended with 195 μl of 25% HCT healthy RBCs in 2% BSA in a PBS suspension, yielding a ~0.025 fraction of HbS RBCs in a 25% HCT blood suspension. Blood was perfused in a two-layer microfluidic device consisting of a blood layer with 21 straight channels, 30 μm wide by 8 μm tall, and a gas layer. Fluorescence images were acquired using a Zeiss Axio Vert microscope enclosed in a 37°C environmental incubator chamber using 20× and 5× objectives (Zeiss APO 20×/0.8-NA and Zeiss 5×/0.16-NA objectives) and imaged using a complementary metal-oxide semiconductor camera (ORCA-Flash4.0LT, Hamamatsu). Three hundred frames at 21 and 0% oxygen were captured for each oxygen tension at ~10 mm from the inlet of the device. Image fluorescence intensity data were processed by averaging the lateral (30-μm-width direction) fluorescence intensity signal across the axial length (flow direction) of the channel using ImageJ to enhance contrast and remove background and custom scripts in MATLAB 2022b for quantification of the processed image stacks. Intensity plots in Fig. 3 (D and G) were each normalized by their respective area under the curve.

### HCT estimation protocol and validation

We estimated the total volume fraction of RBCs in the microfluidic channel using a method described by Roman *et al.* (*79*), whereby the optical density of absorbed light of RBCs is related to the local HCT by counting the cells and multiplying by the RBC volume in the given channel volume (fig. S4). Two standard curves were measured for 21 and 0% oxygen given the differences in absorbance for oxy and deoxy hemoglobin (fig. S4). Similar to Roman *et al.*, we successfully counted cells up to an HCT of 12%. In an attempt to validate and test the dynamic range of our measurement, we flowed packed RBCs into the device. The estimated feed HCT from a sample of the inlet tubing was 57% as measured by a hemacytometer. The tube HCT was estimated using the calibration curve generated in fig. S3A to be 41%. However, because of the known Fahraeus effect, we expect the tube HCT to be lower than the feed HCT for a 20- by 20-μm channel. Therefore, we used the relationship proposed by Pries *et al.* (*32*), which relates the feed HCT to tube HCT, and estimated the tube HCT to be 45%, given a feed of 57%, giving us reasonable confidence in the validity and dynamic range of our measurements.
