# Supplementary material for: Suspension physics govern the multiscale dynamics of blood flow in sickle cell disease
Source: Sci Adv. 2026 Jan 1;12(1):eadx3842. doi: 10.1126/sciadv.adx3842 (PMC12757044; doi:10.1126/sciadv.adx3842)
Supplement: Supplementary file 1 — Figs. S1 to S4 Table S1 Legends for movies S1 to S4 [file sciadv.adx3842_sm.pdf]

Science Advances
AAAS

Supplementary Materials for

Suspension physics govern the multiscale dynamics of blood flow in sickle cell disease

Hannah M. Szafraniec *et al.*

Corresponding author: Philip Pearce, philip.pearce@ucl.ac.uk; David K. Wood, dkwood@umn.edu

Sci. Adv. **12**, eadx3842 (2026)
DOI: 10.1126/sciadv.adx3842

The PDF file includes:

Figs. S1 to S4
Table S1
Legends for movies S1 to S4

Other Supplementary Material for this manuscript includes the following:

Movies S1 to S4

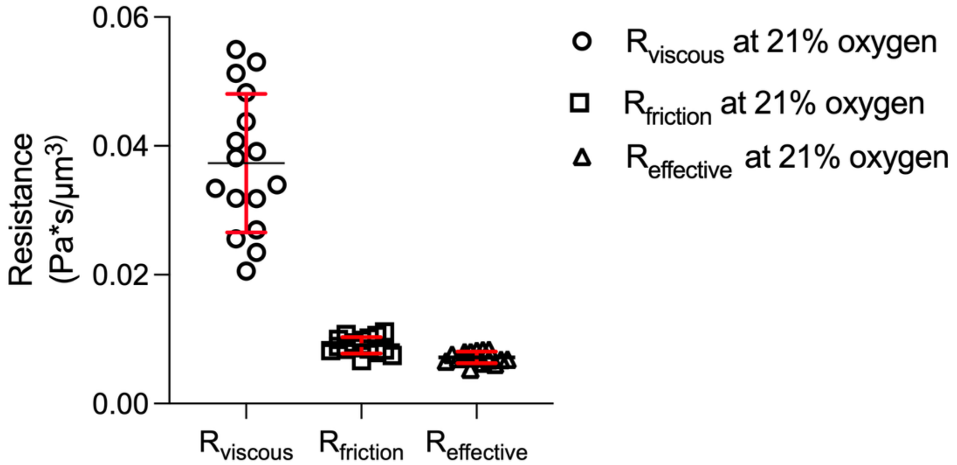

Fig. S1 Raw resistance values of viscous (open circle, 0.037 ± 0.011(SD) Pa s μm^{-3}), friction (open square, 0.0091 ± 0.0013(SD) Pa s μm^{-3}) and effective (open triangle, 0.0072 ± 0.00089(SD) Pa s μm^{-3}) at 21% oxygen. Error bars represent mean ± SD. The viscous and frictional resistance sum in parallel to obtain the effective resistance.

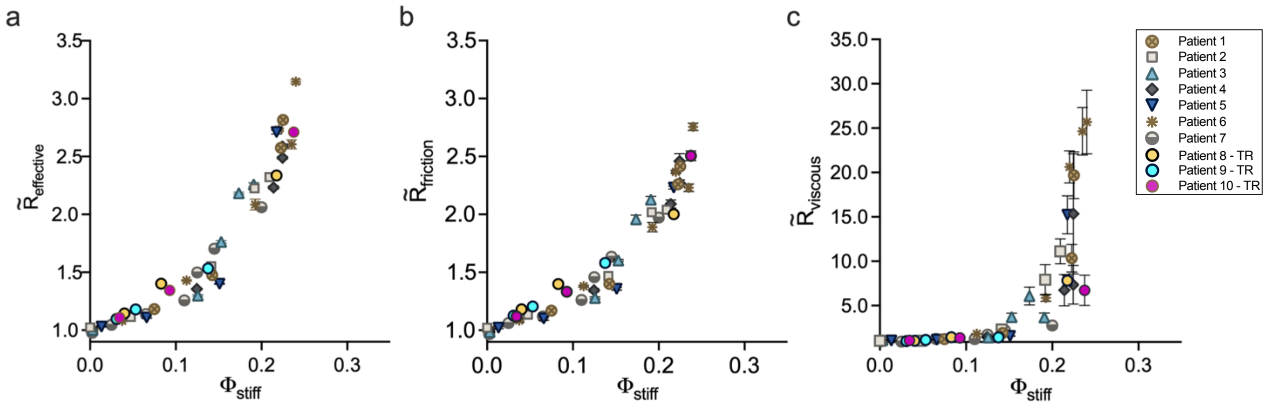

Fig. S2 Effective rheology scaled to volume fraction of stiff RBCs, Φ_{stiff}, where the total volume fraction of RBCs (stiff + deformable) is fixed, $\Phi = 0.25$. **(A)** $\widetilde{R}_{effective}$ plotted as a function of Φ_{stiff}. **(B)** $\widetilde{R}_{friction}$ plotted as a function of Φ_{stiff}. **(C)** $\widetilde{R}_{viscous}$ plotted as a function of Φ_{stiff}. Results shown for n=10 blood samples from SCD donors. Error bars represent plus or minus the standard error of the mean (± SEM) for resistance data gathered for n = 11 independent sampling time points during flow data acquisition. Error bars smaller than the data symbol are not shown.

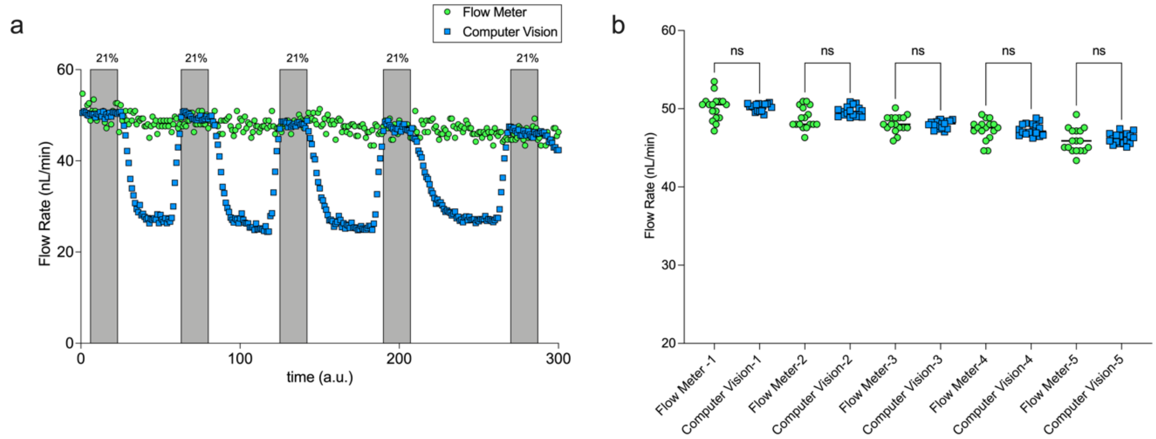

Fig. S3. Flow rate validation between flow meter and computer vision flow rate estimates. **(A)** Temporal data showing flow rate readings from the Fluigent flowmeter (green circle) and flow rate estimates made using the average velocity obtained from computer vision estimates multiplied by the cross-sectional area (blue square). **(B)** Flow rate readings show good agreement when blood is exposed to 21% oxygen. There are no significant differences between the flow meter and computer vision measurements. An ordinary one-way ANOVA with multiple comparisons was performed in Prism v9.5.0 where $p<0.05$ indicates significance.

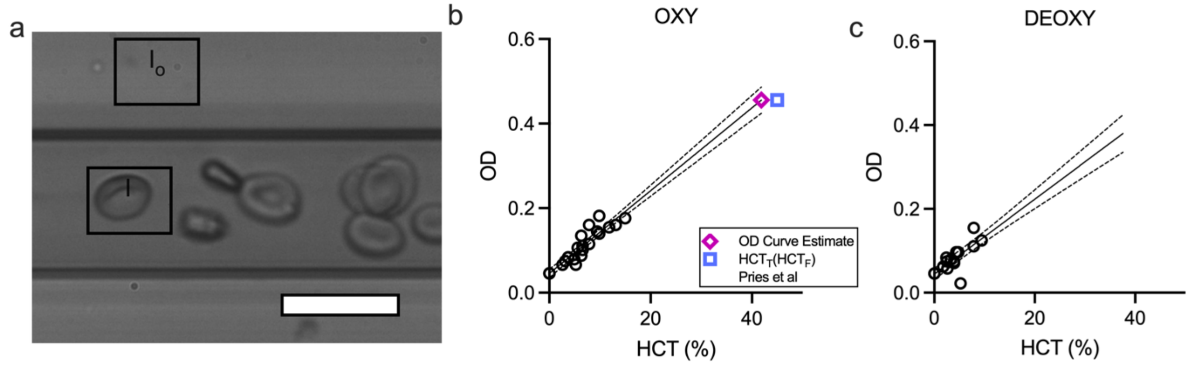

Fig. S4. Hematocrit estimation and validation. **(A)** The optical density of absorbed light of red blood cells is related to the local HCT by counting the cells and multiplying by the mean RBC volume (the mean corpuscular volume is obtained from complete blood count data) in the given channel volume. I is the absorbed light. I_o is the incident light. The channel volume is calculated using the area of the region of interest (black box) multiplied by the channel depth (20 µm). **(B)** Optical density (OD) plotted against estimated hematocrit (HCT (%)) for RBCs under 21% oxygen. Solid line represents the best linear fit to the data, OD = 0.0098 × HCT + 0.045. Dashed line represents 95% confidence interval for the slope (95% CI: 0.0089, 0.011) and y-intercept (95% CI: 0.035, 0.055). Tube HCT of a dense RBC suspension estimated from the calibration curve (pink diamond). Tube HCT predicted from the feed HCT (blue square) by adjusting for the difference in tube and feed HCT for small channels. **(C)** OD plotted against estimated HCT (%) for RBCs under 0% oxygen. Solid line represents the best linear fit to the data, OD = 0.0089 × HCT + 0.044. Dashed line represents 95% confidence interval for the slope (95% CI: 0.0075, 0.010) and y-intercept (95% CI: 0.031, 0.058). Scale bar represents 20 µm. Data were fit using a simple linear regression to obtain the slope and y-intercept, performed in Prism v9.5.0.

Table S1. Patient hematological data. Patient genotype, age, gender/sex, ethnicity, hemoglobin fractions and complete blood count data are included for n=10 blood samples from SCD donors. Acronyms: Hemoglobin S (HbS), hemoglobin A (HbA), hemoglobin F (HbF), hemoglobin A2 (HbA2), white blood cell count (WBC), mean corpuscular volume (MCV), mean corpuscular hemoglobin concentration (MCHC), and red blood cell distribution width (RDW).

Patient ID	Genot-ype	Age	Gender	Ethnicity	HbS (%)	HbA (%)	HbF (%)	HbA2 (%)	WBC (k/uL)	Hg (g/dL)	HCT (%)	MCV (fL)	MCHC (g/dL)	RDW (%)
Patient 1	HbSS	n.d.	Female	Black/African American	89.9	0	7.2	2.9	8.1	10.3	28.9	76	35.6	18.6
Patient 2	HbSS	7	Male	Black/African American	67.9	0	29.1	3	6.2	9.6	28.9	91	33.2	19.1
Patient 3	HbSS	n.d.	Female	Black/African American	75.7	18.5	2.8	3	10.4	7	19.3	87	36.3	23.8
Patient 4	HbSS	12	Male	Black/African American	90.6	0	5.8	3.6	9	8.3	23.7	98	35	22
Patient 5	HbSS	11	Female	Black/African American	80	0	16.5	3.5	6.6	7.7	22.5	69	34.2	16
Patient 6	HbSS	10	Female	Black/African American	80.1	0	16.7	3.2	14.6	8.3	24.2	91	34.3	16.6
Patient 7	HbSS	n.d.	Female	Black/African American	58.5	0	40.1	1.4	4.2	9.5	25.1	113	37.8	15.3
Patient 8	HbSS	15	Male	Black/African American	n.d.	n.d.	n.d.	n.d.	5	10	29.5	101	33.9	17.8
Patient 9	HbSS	n.d.	Male	n.d.	50.6	35.4	11.2	2.8	8.95	n.d.	27.3	97.5	34.8	n.d.
Patient 10	HbSS	12	Male	Black/African American	89.2	0	7.4	3.4	11.1	9.8	27.8	96	35.3	17.8

Movie S1 Video of spatial distribution of fluorescent signal under 21% oxygen for fluorescent SS RBCs. Videos acquired at 20 fps and 20x magnification. Playback speed at 20 fps.

Movie S2 Video of spatial distribution of fluorescent signal under 0% oxygen for fluorescent SS RBCs. Videos acquired at 20 fps and 20x magnification. Playback speed at 20 fps.

Movie S3 Video of actively marginating polystyrene beads in 25% hematocrit blood at 21% oxygen. Video acquired at 400 fps and 10x magnification. Playback speed at 40 fps.

Movie S4 Video of axial variations in hematocrit at 0% oxygen. Video acquired at 400 fps and 5x magnification. Playback speed at 40 fps.
